# Prevalence of uncontrolled blood pressure in Meknes, Morocco, and its associated risk factors in 2017

**DOI:** 10.1371/journal.pone.0220710

**Published:** 2019-08-09

**Authors:** Touria Essayagh, Meriem Essayagh, Abderrahmane El Rhaffouli, Mohammed Khouchoua, Germain Bukassa Kazadi, Asmae Khattabi, Sanah Essayagh

**Affiliations:** 1 Laboratoire Sciences et Technologies de la Santé, Institut Supérieur des Sciences de la Santé, Université Hassan 1^er^, Settat, Morocco; 2 Ecole Nationale de Santé Publique, Rabat, Morocco; 3 Faculté de Médecine et de Pharmacie, Université Mohammed V, Rabat, Morocco; 4 Délégation de la Santé, Meknès, Morocco; 5 Department of Indigenous Services Canada/Government of Canada, Health Surveillance and Assessment Unit, First Nations and Inuit Health, Saskatchewan Region, Regina, Sk Canada; 6 Laboratoire Agroalimentaire et Santé, Faculté des Sciences et Techniques, Université Hassan 1^er^, Settat, Morocco; Makerere University School of Public Health, UGANDA

## Abstract

**Background:**

Uncontrolled high blood pressure (UBP) can lead to various cardiovascular complications causing an estimated nine million deaths per year worldwide. In Meknes, epidemiologic data on UBP are scarce, depriving programs from evidence-based information that would allow a better management of hypertension. Hence, we aimed to assess UBP prevalence in hypertensive patients treated in Meknes and identify UBP-associated risk factors.

**Methods:**

Between November and December 2017, we conducted a cross-sectional study enrolling 922 hypertensive patients managed at Meknes’s primary health care facilities using the multistage sampling method. We interviewed patients face to face to collect their socio-demographic-characteristics, lifestyle behaviours, clinical parameters and the triad care system-patient-physician. Another questionnaire was self-administered by physicians to characterize therapeutic inertia. A multivariate logistic regression analysis highlighted the risk factors associated with UBP.

**Results:**

UBP prevalence was 73% with a mean age of 61±11 years (mean±standard deviation) and a male/female ratio of 1/3. Risk factors associated with UBP were: therapeutic inertia (adjusted odds ratio to other variables (AOR) = 18.2, 95% CI [8.35–39.84]), drug non-adherence (AOR = 1.8, 95% CI [1.07–3.04]), obesity/overweight (AOR = 1.6, 95% CI [1.03–2.58]), unemployment (AOR = 1.9, 95% CI [1.09–3.01]), low income (AOR = 2.6, 95% CI [1.01–6.86]), family history of hypertension (AOR = 1.5, 95% CI [1.07–2.08]) and male sex (AOR = 1.6, 95% CI [1.04–2.58]).

**Conclusion:**

UBP prevalence is high in Meknes. Prevention should firstly focus on raised awareness of hypertensive patients’ self-care management. Secondly, health professionals should better comply to the guidelines of anti-hypertensive treatments. Lastly, health professionals should frequently be reminded to reach therapeutic goals to overcome therapeutic inertia.

## Introduction

Abnormally high blood pressure values define hypertension. Hypertension is a major risk factor and the cause of an estimated nine million deaths per year worldwide [1–3]. In 2000, hypertension affected an estimated 972 million people, including 639 million in developing countries [4]. Projections based on these data suggest that the number will increase by 60% in 2025 [5]. Low- and middle-income countries account for more than three quarters of global deaths related to cardiovascular diseases according to the World Health Organization [1, 6]. Premature deaths, personal and family disruption, loss of income, and high health care costs associated with hypertension are a significant economic burden for families, communities, and public health system with 57 million disability-adjusted life year (DALY) at the global level and 658.301 DALY in Morocco [7]. Due to its chronicity and the need for lifespan treatment, hypertension is hence a factor of poverty [8].

The Moroccan budget allocated to tackle hypertension rose from 33 million dirhams in 2010 to 45 million dirhams in 2014 [7]. A first Moroccan national survey was conducted in 2000 on a sample of 1802 participants and revealed that hypertension had an estimated prevalence of 33% among people aged 20 and over [9]. According to regional records, the 46 primary health care facilities (PHCF) of the prefecture of Meknes followed approximately 15515 hypertensive patients in 2016, 1793 of whom had complications. Altogether, it appears that data on hypertension in Morocco are still very scarce. A better knowledge of the current characteristics of the hypertensive population would highly improve the clinical management of the disease and its cardiovascular complications. A multi-center epidemiological trial of hypertension in North Africa conducted between September 2008 and January 2009 involving 28.500 patients showed that 45% of the population were known as hypertensive and 64% of those individuals had uncontrolled blood pressure (UBP) [7, 10]. The benefit of antihypertensive treatment should correlate with the decline in blood pressure. UBP (systolic blood pressure greater than 140 mmHg and / or diastolic blood pressure greater than 90 mmHg after treatment) is often complicated by serious cardiovascular events for which it multiplies the risk of death [11, 12]. To date, there is no available data in Morocco regarding the prevalence of UBP and its associated determinants. The objective of this study is to assess the prevalence of UBP in hypertensive patients followed at PHCF in Meknes and to identify its associated risk factors.

## Methods

### Study design and population

We performed a cross-sectional study with descriptive and analytical purposes from November 1^st^ to December 31^st^ of 2017. We based sampling on a stratified two-stage survey. The primary unit consisted of the primary health care centers. The secondary unit consisted of hypertensive patients undergoing a medical treatment to correct high blood pressure for at least six months, residing in Meknes and attending one of the six health centers where the prevalence of hypertension is the highest and who have agreed to participate in the survey. We selected these six centers according to the reported prevalence of hypertension at the level of each PHCF and after weighting according to the urban (85%) or rural (15%) origin of the patients.

In each primary unit selected, we performed a random draw of the patient order numbers to determine the secondary unit. This technique facilitated the survey and reduced costs.

### Sample size determination

Based on a 95% confidence interval, a prevalence of UBP estimate of 50%, setting the margin of error at 5%, achieving 20% inflation and a cluster effect of 2, we have set the minimum sample size to 922 hypertensive patients. We excluded pregnant women and individuals with mental disability from the study population. We also excluded seven patients presenting with secondary high blood pressure as documented in their medical records.

### Data collection

We used two questionnaires: we applied the first standardized questionnaire to eligible patients who answered questions during a face-to-face individual interview. We collected the following data: socio-demographic background, perception of the disease by the patient, lifestyle and behavioural habits, clinical parameters and the triad care system-patient-physician. The second questionnaire was self-administered by physicians and focused on therapeutic inertia.

### Operational definitions

We measured the blood pressure on a patient sitting his back against a chair at the end of the interview *i*.*e*. after 20 minutes of rest, his arm supported so that it was at the height of the heart, his feet on the ground while avoiding crossed legs and ensuring he was not speaking during the measurement. We used an electronic blood pressure monitor (MicroLife Pro M with an accuracy of ±3 mmHg) with a cuff sized for the age of the patient. We performed a preliminary blood pressure measurement on each arm to determine which had the highest blood pressure value. We then monitored two successive measurements of the blood pressure on the arm with the highest blood pressure with an interval of one minute between them. We defined the average of these last two measurements as the patient’s average arterial pressure. To ascertain UBP, we collected arterial pressure measurements from the patient's medical chart and we recorded the average of the last three arterial pressure measured over the preceding six months which include one done during this study. UBP was classified according to the criteria of the European Society of Hypertension and the European Society of Cardiology (ESH/ESC) guidelines [13]. According to these guidelines, UBP was identified in the treated general population when the systolic blood pressure (SBP) was greater than or equal to 140 mmHg whereas the diastolic blood pressure (DBP) was greater than or equal to 90 mmHg. High blood pressure was noted as controlled if the SBP was less than 140 mmHg and / or the DBP less than 90 mmHg. In diabetic patients, UBP was identified if, after treatment, the SBP was greater than or equal to 140 mmHg and / or the DBP greater than or equal to 85 mmHg. It was noted as controlled if the SBP was less than 140 mmHg and / or the DBP less than 85 mmHg. In patients with renal failure diagnosed at the end stage of kidney disease, UBP was acknowledged if, after treatment, the SBP was greater than or equal to 130 mmHg and / or the DBP greater than or equal to 90 mmHg. It was noted as controlled if the SBP was less than 130 mmHg and / or a DBP less than 90 mmHg. The level of hypertension was classified according to the criteria of ESH/ESC guidelines as [13]: grade I, SBP 140–159 mmHg and/or DBP 90-99mmHg; grade II, SBP 160–179 mmHg and/or DBP 100–109 mmHg; grade III, SBP 180 mmHg and/or DBP 110 mmHg; isolated systolic hypertension, SBP ≥140 mmHg and DBP<90mmHg. We defined obesity according to the WHO classification of the body mass index (BMI); abdominal obesity was defined by waist circumference measurement (males⩾ 102 cm; females⩾ 88 cm). Cardiovascular risk was split into two categories: high or low. Drug adherence was assessed by the Girerd Drug Adherence Evaluation Test [4, 14]. This test gathers six questions to which the patient must answer yes or no; the questions asked are included in the patient’s questionnaire in the section “compliance with treatment” “S1 Questionnaire”. Patients with a yes response number greater than 3 were rated poorly observing, patients with a yes response number between 1 and 2 were rated moderately observant and those with zero yes were rated good observant. The therapeutic inertia was evaluated by the Okonofua modified method as described previously [15]. Briefly, we considered that there was therapeutic inertia when there was no change in treatment despite an abnormal blood pressure value during two successive consultations and a minimum of one month between these two consultations and no change in treatment in the last 3 months. The 3 months with no change in treatment correspond to the minimum period of balanced patient monitoring, which is often the time for renewal visits for antihypertensive treatment.

### Data management and statistical analysis

We maintained the consistency of the compiled data by editing and checking for duplicates. We removed duplicates before coding and analysis using Epi-Info-7.2.0.1. We calculated frequencies and percentages to identify the distribution of the socio-demographic characteristics. All tests were two-tailed, and we considered a p value < 0.05 to be statistically significant.

We expressed the results as mean ± standard deviation for quantitative variables or as percentages for qualitative variables. To address the objectives of the study, we performed a bivariate analysis to identify the factors associated with UBP in hypertensive patients and a multivariate analysis to neutralize the confounding factors and determine the simultaneous effect of independent variables on the poor control of HBP. We used logistic regression in the bivariate and multivariate analysis; we fitted into multivariable logistic regression any variable with a p-value up to 0.2 in the bivariate analysis. We used a backward stepwise logistic regression procedure to determine the final model. We estimated the association between each assumed risk factor and UBP using the adjusted odds ratio and its 95% confidence interval.

### Ethical considerations

The ethical review board of the Faculty of Medicine of Rabat in Morocco reviewed and approved the study protocol. We obtained consent from all participants after detailed explanation of the research purpose and assurance of maintaining privacy and confidentiality. Thus, we rigorously implemented all precautions to respect anonymity and confidentiality of the patient’s information by using a coding system. Data were accessible only to the research team. A list containing the patient’s code, his surname and first name, his contact information was established; the coordinator of the study could, in case of needing further information, identify the patient from his code, by his name and surname. No one else, apart from the study coordinator, had access to this list.

## Results

### 1. Results socio-demographic and economic characteristics

During the study period, 922 hypertensive patients were enrolled of whom 675 had uncontrolled hypertension corresponding to a prevalence of 73%. The male/female ratio was 1/3 and the average age was 61±11 years with a range from 30 to 100 years. Married individuals accounted for 63% of cases and illiterates accounted for 73% of cases. More than 88% of the cases were unemployed with 79% having a monthly income of less than or equal to 1500 MAD ($150) and 52% had a Moroccan medical assistance plan (RAMED) “Table 1”.

**Table 1:** Socio-demographic and economic characteristics of hypertensive patients followed up at primary health care facilities, Meknes, Morocco, 2017.

Quantitative variables		n = 922	Uncontrolled BP	Controlled BP	p-value
Patients’ total no. (%)		922 (100)	675 (73.2)	247 (26.8)	
Age in years±sd		61.4±10.8	61.4±10.5	61.6±11.9	0.96
**Qualitative variables no. (%)**					
Age group in years					0.03
	≥80	68 (7.4)	42 (61.8)	26 (38.2)	
	[70–79]	159 (17.2)	118 (74.2)	41 (25,8)	
	[60–69]	333 (36.1)	256 (76.9)	77 (23.1)	
	[50–59]	255 (27.6)	190 (74.5)	65 (25.5)	
	[40–49]	94 (10.2)	62 (66.0)	32 (34.0)	
	[30–39]	13 (1.4)	7 (53.8)	6 (46.2)	1
Sex	Male	205 (22.2)	160 (78.0)	45 (22.0)	0.07
	Female	717 (77.8)	515 (71.8)	202 (28.2)	1
Area of residence					
	Urban	781 (84.7)	574 (73.5)	207 (26.5)	0.64
	Rural	141 (15.3)	101 (71.6)	40 (28.4)	1
Marital status					0.71
	Widower	253 (27.4)	192 (75.9)	61 (24.1)	
	Divorced	58 (6.3)	41 (70.7)	17 (29.3)	
	Single	26 (2.8)	19 (73.1)	7 (26.9)	
	Married	585 (63.4)	423 (72.3)	162 (27.7)	1
Education					0.45
	Illiterate	677 (73.4)	490 (72.4)	187 (27.6)	
	Elementary	159 (17.2)	125 (78.6)	34 (21.4)	
	Middle school	41 (4.4)	28 (68.3)	13 (31.7)	
	High school	34 (3.7)	25 (73.5)	9 (26.5)	
	College	11 (1.2)	7 (63.6)	4 (36.4)	1
Occupation					
	No	814 (88.3)	605 (74.3)	209 (25.7)	0.04
	Yes	108 (11.7)	70 (64.8)	38 (35.2)	
Monthly income per household ($)					0.13
	<150	728 (79.0)	539 (74.0)	189 (26.0)	
	[150–200[	96 (10.4)	69 (71.9)	27 (28.1)	
	[200–300[	57 (6.2)	42 (73.7)	15 (26.3)	
	[300–500[	17 (1.8)	13 (76.5)	4 (23.5)	
	≥500	24 (2.6)	12 (50.0)	12 (50.0)	1
Health insurance plan					0.68
	Without	189 (20.5)	140 (74.1)	49 (25.9)	
	RAMED	480 (52.0)	344 (71.7)	136 (28.3)	
	Other	28 (3.0)	22 (78.6)	6 (21.4)	
	Mutual	225 (24.4)	169 (75.1)	56 (24.9)	1

RAMED: Moroccan medical assistance plan, sd: standard deviation. For qualitative variables, the Pearson chi-2 test estimated the association between the dependent variable and the independent variables when the conditions were valid. For the quantitative variables, we used a comparison test of two means; p-value was considered significant when it was less than 0.05.

### 2. Perception of the disease by the patient

Among the 922 hypertensive subjects we enrolled, only 69 (7%) had a general knowledge of hypertension. The repartition by level of knowledge on this pathology showed that 57 (6%) knew the clinical signs of the disease, 423 (46%) knew its means of prevention and 264 (29%) knew its complications.

### 3. Lifestyle and behavioural characteristics

Analysis of the behavioural data showed that 716 hypertensive subjects (77%) had between one and two risk factors. Physical activity was unsatisfactory in 243 cases (26%). The sodium diet was reported in 162 hypertensive patients (18%) and the non- adherence with diet and lifestyle habits was recorded in 826 cases (90%) “Table 2”.

**Table 2:** Behavioural characteristics of hypertensive patients monitored at primary health care facilities, Meknes, Morocco, 2017.

Qualitative variables no. (%)		n = 922	Uncontrolled BP (n = 675)	Controlled BP (n = 247)	p-value
Tobacco use					0.88
	Current smoker	21 (2.3)	15 (71.4)	6 (28.6)	
	Former smoker	58 (6.3)	44 (75.9)	14 (24.1)	
	Non-smoking	843 (91.4)	616 (73.1)	227 (26.9)	1
Alcohol consumption					0.02
	Frequent	21 (2.3)	20 (95.2)	1 (4,8)	
	Occasional	14 (1.5)	8 (57.1)	6 (42.9)	
	Never	887 (96.2)	647 (72.9)	240 (27.1)	1
Physical activity					
	Unsatisfactory	243 (26.4)	176 (72.4)	67 (27.6)	0.74
	Satisfactory	679 (73.6)	499 (73.5)	180 (25.5)	1
Stress					0.57
	Intense	284 (30.8)	204 (71.8)	80 (28.2)	
	Intermediate	489 (53.0)	357 (73.0)	132 (27.0)	
	None	149 (16.2)	114 (76.5)	35 (23.5)	1
Salty diet					0.47
	Salty	162 (17.6)	117 (72.2)	45 (27.8)	
	Semi-salted	713 (77.3)	520 (72.9)	193 (27.1)	
	Without	47 (5.1)	38 (80.9)	9 (19.1)	1
Duration of high blood pressure in years					
	>5	464 (50.3)	353 (76.1)	111 (23.9)	0.04
	≤5	458 (49.7)	322 (70.3)	136 (29.7)	1
Family history of high BP					
	Yes	485 (52.6)	371 (76.5)	114 (23.5)	0.01
	No	437 (47.4)	304 (69.6)	133 (30.4)	1
Self-monitoring					
	No	810 (87.9)	593 (73.2)	217 (26.8)	1.0
	Yes	112 (12.1)	82 (73.2)	30 (26.8)	1
Lifestyle and dietary advice					
	No	826 (89.6)	602 (72.9)	224 (27.1)	0.50
	Yes	96 (10.4)	73 (76.0)	23 (34.0)	1
Annual biological check-up					
	No	727 (78.9)	546 (75.1)	181 (24.9)	0.01
	Yes	195 (21.1)	129 (66.1)	66 (33.9)	1
Number of risk factors					0.22
	≥3	185 (20.1)	134 (72.4)	51 (27.6)	
	1–2	716 (77.6)	529 (73.9)	187 (26.1)	
	None	21 (2.3)	12 (57.1)	9 (42.9)	1

BP: blood pressure. For qualitative variables, the Pearson chi-2 test estimated the association between the dependent variable and the independent variables when the conditions were valid; p-value was considered significant when it was less than 0.05.

### 4. Clinical features

**Fig 1:**
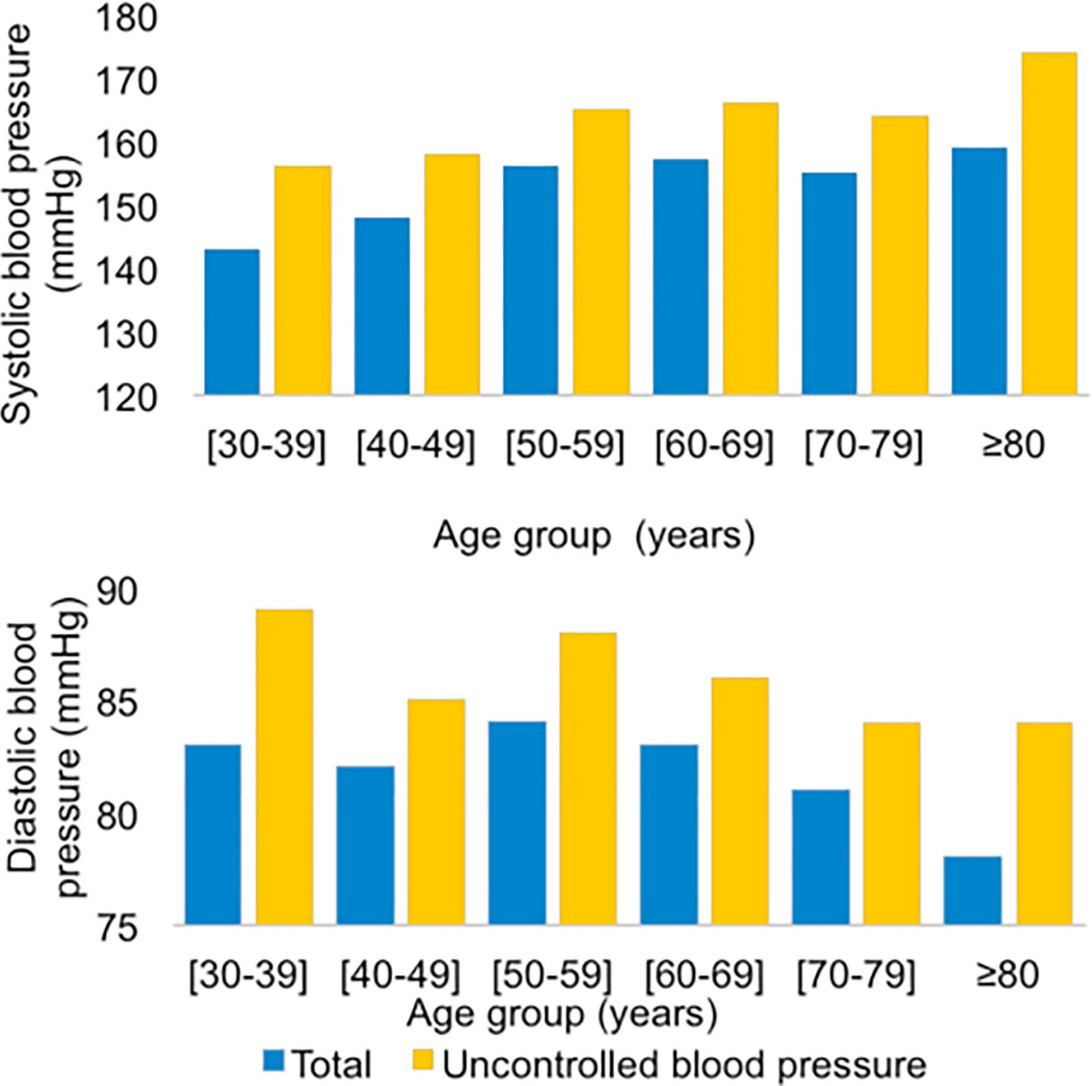
Distribution of means of systolic and diastolic blood pressure values by age groups, Meknes, Morocco, 2017.

Mean SBP was 155.7±22.7 mmHg while DBP was 82.5±12.6 mmHg in the hypertensive patients recruited for the study. The data from the study showed that blood pressure increased gradually from the age of 40 years. As early as age 50, we found that SBP continues to increase as DBP gradually decreases. The presence of comorbidity associated with hypertension was found in 577 cases (63%) with a predominance of diabetes (56%) “Fig 1”. In terms of health characteristics, overweight and obesity were predominant in 782 (85%) hypertensive patients; the same is true for abdominal obesity in 717 hypertensive individuals (78%) “Table 3”. During the study period, 606 hypertensive patients had a high cardiovascular risk, 55% of whom did not have medical management of risk factors.

**Table 3:** Clinical features of hypertensive patients at primary health care facilities, Meknes, Morocco, 2017.

Qualitative variables no. (%)		n = 922	Uncontrolled BP (n = 675)	Controlled BP (n = 247)	p-value
Comorbidity					
	Yes	577 (62.6)	421 (73.0)	156 (27.0)	0.82
	No	345 (37.4)	254 (73.6)	91 (26.4)	1
Diabetes					
	Yes	515 (55.8)	378 (73.4)	137 (26.6)	0.88
	No	407 (44.1)	297 (73.0)	110 (27.0)	1
Cardiovascular disease					
	Yes	87 (9.4)	60 (69.0)	27 (31.0)	0.37
	No	835 (90.6)	615 (73.7)	220 (26.3)	1
Dyslipidemia					
	Yes	146 (15.8)	105 (72.0)	41 (28.1)	0.70
	No	776 (84.2)	570 (73.5)	206 (26.5)	1
Kidney disease					
	Yes	24 (2.6)	13 (54.2)	11 (45.8)	0.03
	No	898 (97.5)	662 (73.7)	236 (26.3)	1
Overweight/obesity					
	Yes	782 (84.8)	580 (74.2)	202 (25.8)	0.12
	No	140 (15.2)	95 (67.9)	45 (32.1)	1
Abdominal obesity					
	Yes	717 (77.7)	519 (72.4)	198 (27.6)	0.28
	No	205 (22.2)	156 (76.1)	49 (23.9)	1
Waist-hip ratio					
	Android obesity	716 (77.6)	519 (72.5)	197 (27.5)	0.32
	Gynoid obesity	206 (22.3)	156 (75.7)	50 (24.3)	1

BP: blood pressure. For qualitative variables, the Pearson chi-2 test estimated the association between the dependent variable and the independent variables when the conditions were valid; p-value was considered significant when it was less than 0.05.

### 5. Triad care system-patient-physician

Among the 922 hypertensive patients surveyed, 665 (72%) had an average satisfaction with the health care system. Poor adherence with the drug was reported in 244 cases (27%). The patient-physician relationship was considered to be poor in 367 (40%) hypertensive subjects “Table 4”.

**Table 4:** Characteristics of the triad care system-patient-physician amongst hypertensive patients followed at the level of primary health care facilities, Meknes, Morocco 2017.

Qualitative variables no. (%)		n = 922	Uncontrolled BP (n = 675)	Controlled BP (n = 247)	p-value
Relationship with the health care system					0.92
	Bad	36 (3.9)	26 (72.2)	10 (27.8)	
	Intermediate	665 (72.1)	485 (72.9)	180 (27.1)	
	Good	221 (23.9)	164 (74.2)	57 (25.8)	1
Drug adherence					<0.01
	Bad	244 (26.5)	172 (70.5)	72 (29.5)	
	Intermediate	593 (64.3)	453 (76.4)	140 (23.6)	
	Good	85 (9.2)	50 (58.8)	35 (41.2)	1
Physician-patient relationship					0.40
	Bad	367 (39.8)	274 (74.7)	93 (25.3)	
	Intermediate	537 (58.2)	386 (71.9)	151 (28.1)	
	Good	18 (2.0)	15 (83.3)	3 (16.7)	1
Therapeutic inertia					
	Yes	238 (25.8)	231 (97.0)	7 (3.0)	<0.01
	No	684 (74.2)	444 (64.9)	240 (35.1)	1
Medical management of risk factors					
	No	547 (59.3)	396 (72.4)	151 (27.6)	0.49
	Yes	375 (40.7)	279 (74.4)	96 (25.6)	1
Duration of treatment in months					
	>6	836 (90.7)	622 (74.4)	214 (25.6)	0.01
	≤ 6	86 (9.3)	53 (61.6)	33 (38.4)	1
Number of molecules					0.49
	Monotherapy	829 (89.9)	604 (72.9)	225 (27.1)	
	Bitherapy	90 (9.8)	68 (75.6)	22 (24.4)	
	Tritherapy	3 (0.3)	3 (100.0)	0 (0.0)	1
Type of treatment					
	Generic	879 (95.3)	646 (73.5)	233 (26.5)	0.38
	Brand/trademark	43 (4.7)	29 (67.4)	14 (32.6)	1

For qualitative variables, the Pearson chi-2 test estimated the association between the dependent variable and the independent variables when the conditions were valid; p-value was considered significant when it was less than 0.05.

### 6. Characteristics of antihypertensive treatment

Among the 922 hypertensive patients enrolled in the study, 836 (91%) had been on the same treatment for more than six months. 829 (90%) were on monotherapy and 90 (10%) on dual therapy. Generic treatment was predominant in 879 (95%) hypertensive patients “Table 4”.

### 7. Uncontrolled blood pressure

Among the 922 hypertensive patients included in the study, 675 had an UBP, which means a prevalence of 73%. Their mean age was 61±10 years with extremes ranging from 30 to 98 years and a sex ratio male/female of 1/3. Analysis of the socio-economic data showed that out of 675 hypertensive subjects with UBP, 89% were unemployed and 80% had a monthly income per household of less than 150$ “Table 1”.

Behavioural data analysis revealed that of the 675 UBP, 89% did not comply with lifestyle and dietary measures. The lack of annual physical exam and check-up amongst patients included in the study, an essential element in the management of hypertension, was noted in 81% of patients with UBP, *i*.*e*. 546 hypertensive patients. Similarly, 73% of these hypertensive patients did not perform blood pressure self-measurement, corresponding to 593 hypertensive patients.

The distribution of hypertensive subjects with UBP by level of blood pressure revealed that 48% had isolated systolic blood pressure, 16% had blood pressure grade I, 7% had blood pressure grade II and 2% had blood pressure grade III.

Analysis of the clinical data of 675 hypertensive subjects with UBP showed that 2% had chronic kidney disease “Table 3” and 67% followed a monodrug therapy “Table 4”. Among 589 hypertensive patients with high cardiovascular risk and for whom hypertension was uncontrolled, 45% did not have a good management of risk factors.

Therapeutic inertia was prevalent in 34% of the study population, which means 231 UBP for whom the antihypertensive treatment was not intensified or modified during the last two consultations “Table 4”.

### 8. Bivariate analysis

We enrolled 922 hypertensive patients included in the study; 675 (73%) of them had UBP. The cut-off p-value after the bivariate analysis was set at p<0.2. Accordingly, we identified thirteen factors associated with UBP: 1. therapeutic inertia (p = 0.01), 2. treatment longer than six months (p = 0.01), 3. absence of an annual physical exam (p = 0.01), 4. family history of hypertension (p = 0.01), 5. drug non-adherence (p = 0.01), 6. alcohol consumption (p = 0.02), 7. age (p = 0.04), 8. non-employment (p = 0.04), 9. a duration of high blood pressure of more than 5 years (p = 0.04), 10. renal disease (p = 0.04), 11. male sex (p = 0.07),12. low monthly income per household (p = 0.13), and 13. overweight/obesity (p = 0.12) “Table 5”.

**Table 5:** Multivariate analysis (odds ratio, p-value) of uncontrolled blood pressure in hypertensive patients followed at primary health care facilities, Meknes, 2017.

Qualitative variables			Bivariate analysis		Multivariate analysis complete model		Multivariate analysis final model	
			COR 95%CI	p-value	AOR 95%CI	p-value	AOR 95%CI	p-value
Male/female ratio			1.4 [0.96–2.01]	0.07	1.6 [1.00–2.52]	0.05	1.6 [1.04–2.58]	0.03
Age in years				0.04				
≥80			1.4 [0.41–4.57]	0.59	1.4 [0.30–6.30]	0.66	1.4 [0.31–6.46]	0.65
[70–79]			2.5 [0.78–7.76]	0.12	2.2 [0.51–9.38]	0.28	2.2 [0.51–9.41]	0.29
[60–69]			2.8 [0.93–8.73]	0.06	2.8 [0.70–11.84]	0.14	2.9 [0.69–11.93]	0.14
[50–59]			2.5 [0,81–7.72]	0.10	2.7 [0.66–11.20]	0.16	2.6 [0.63–10.88]	0.18
[40–49]			1.7 [0.51–5.35]	0.39	1.6 [0.38–7.03]	0.50	1.5 [0.35–6.66]	0.56
[30–39]				1		1		1
Unemployment			1.6 [1.03–2.40]	0.04	1.8 [1.06–2.95]	0.02	1.8 [1.09–3.01]	0.02
Monthly income per household in $								
	<150		2.8 [1.25–6.45]	0.01	2.8 [1.07–7.34]	0.03	2.6 [1.01–6.86]	0.04
	[150–200[		2.5 [1.02–6.37]	0.04	2.3 [0.80–6.68]	0.11	2.1 [0.74–6.22]	0.15
	[200–300[		2.8 [1.03–7.55]	0,04	2.3 [0.71–7.27]	0.16	2.1 [0.66–6.73]	0.20
	[300–500[		3.2 [0.81–12.86]	0.09	2.9 [0.63–13.45]	0.17	2.8 [0.61–13.04]	0.18
	≥500			1		1		1
Alcohol consumption								
	Frequent		7.4 [0.99–55.55]	0.05	7.9 [0.97–69.47]	0.05	7.7 [0.95–62.06]	0.06
	Occasional		0.4 [0.17–1.44]	0.19	0.2 [0.06–0.93]	0.04	0.2 [0.06–0.93]	0.04
	Never			1		1		1
Obesity/overweight			1.4 [0.92–2.00]	0.12	1.5 [0.96–2.45]	0.07	1.6 [1.03–2.58]	0.04
Annual biological check-up			1.5 [1.09–2.17]	0.01	1.4 [0.93–2.03]	0.10	1.4 [0.97–2.08]	0.07
Familial history			1.4 [1.06–1.90]	0.01	1.4 [1.04–2.02]	0.03	1.5 [1.07–2.08]	0.02
Duration of high blood pressure for more than 5 years			1.3 [1.00–1.79]	0.04	1.3 [0.93–1.82]	0.12	*****	*****
Duration of treatment less than 6 months			1.8 [1.14–2.87]	0.01	1.4 [0.84–2.34]	0.20	*****	*****
Kidney disease			0.4 [0.18–0.95]	0.04	0.5 [0.19–1.29]	0.15	*****	*****
Drug adherence								
	Bad		1.7 [1.00–2.79]	0.04	1.4 [0.78–2.46]	0.26	1.4 [0.80–2.52]	0.21
	Intermediate		2.3 [1.41–3.63]	<0.01	1.7 [1.01–2.89]	0.04	1.8 [1.07–3.04]	0.02
	Good			1		1		1
Therapeutic inertia			17.8 [8.27–38.45]	<0.01	18.1 [8.30–39.6]	<0.01	18.2 [8.35–39.84]	<0.01

COR: crude odds ratio, AOR: adjusted odds ratio, CI: confidence interval

### 9. Multivariate analysis

After adjusting for the other variables, the following factors were identified as predictor factors of UBP in hypertensive patients treated at Meknes’ PHCF: 1. therapeutic inertia (adjusted odd ratio (AOR) = 18.2, 95% CI [8.35–39.84]), 2. the low income per household (AOR = 2.6, 95% CI [1.01–6.86]), 3. non-employment (AOR = 1.8, 95% CI) [1.09–3.01]), 4. drug non-adherence (AOR = 1.8, 95% CI [1.07–3.04]), 5. the male sex (AOR = 1.6, 95% CI [1.04–24.88]), 6. overweight/obesity (AOR = 1.6, 95% CI [1.03–2.58]), 7. and the family history of hypertension (AOR = 1.5, 95% CI [1.07–2.08]) “Table 5".

## Discussion

In the current study, the prevalence of UBP was 73%. To our knowledge, there is no previous national study estimating nor analysing of UBP prevalence amongst hypertensive patients followed at PHCF in Morocco. In countries where the socio-economic and demographic statuses of the population are close to those existing in Morocco, the prevalence of UBP was similar. Indeed, in 2012, in Oran, Algeria, the prevalence of UBP was 69% when analysing a sample of 253 hypertensive patients [11]. In Blidia, Algeria, between 2014 and 2016, the analysis of a sample of 3622 hypertensive patients revealed that the prevalence of UBP was 70% [16].

In a multi-center survey of 12933 known hypertensive patients residing in Tunisia, Algeria or Morocco, the prevalence of UBP was 64% [7, 10]. However, the results did not report data specific to Morocco. In developed countries, the prevalence of UBP was lower. In France, between 2014 and 2016, the prevalence of UBP in a sample of 2169 hypertensive patients was 45% [17].

Moreover, in our work, age increased the risk for the frequency of UBP. Our study is consistent with results reported and published from studies conducted in Algeria and Burkina Faso [11, 18] as well as results reported from a study conducted in the USA [19]. The 1948 Framingham survey, conducted on a sample of 5000 people, showed that SBP increased linearly with age until age 80–90, while DBP increased until age 80–90. It has been reported that starting at age 50, there is a loss of elasticity of the arterial walls [19, 20] that would account for the continuous increase of SBP observed. We report the same observation in our study. Anti-hypertensive treatment administered to hypertensive patients had a preferential action on DBP and was less effective on SBP. This is why in a large proportion of elderly people undergoing treatment, systolic-diastolic hypertension progresses to isolated systolic hypertension [19]. This could explain the predominance of isolated SBP in our survey.

In our findings, males are more likely to be associated with UBP. This finding is similar to the results of the cross-sectional study conducted in Congo on a sample of 620 hypertensive patients [21]. However, different results were obtained in a study conducted in Zimbabwe and Malaysia that have been in contrast with our current findings [22, 23].

Lack of employment and low monthly income per household are more likely associated with UBP. This result has been reported in other studies, particularly in Burkina-Faso, Zimbabwe, China and other countries [18, 23–29].

Illiteracy rates at a high-level among hypertensive patients; it was predominant in our study and was significantly associated with UBP. This is similar to the results reported by the multi-center study conducted in Tunisia, Algeria and Morocco [10].

With regard to the medical plan, among the 922 hypertensive patients, 480 (52%) have a RAMED card and 225 (24%) a health care plan. However, more than 727 (79%) did not get their annual physical exam and check-up necessary for systematic medical monitoring including 423 (46%) patients with UBP. This study was an opportunity to raise the awareness of the benefits offered by the RAMED program to cover the most fragile population as well as providing information about the complications of hypertension. Another reason could be adding appointments that are widely spaced in time, which can range from 3 to 6 months in order to benefit from a specialized consultation.

In our study, occasional consumption of alcohol seemed to be a protective factor against uncontrolled hypertension. Indeed, multiple reports have demonstrated that regular and moderate consumption of alcohol is associated with a decrease in the overall risk of cardiovascular disease. This decrease is due to the beneficial effects of wine on lipoproteins and coagulation factors. However, it is important to remember that frequent alcohol consumption has no positive effect on blood pressure figures, rather, it is associated with increased hypertension [30].

Although a sedentary lifestyle plays an important role in UBP, this has not emerged in our study. It is the same for the excessive consumption of salt. Indeed, many studies have criminalized the sodium diet as a risk factor associated with UBP [11, 31] or even the appearance of cardiovascular events [32, 33]. A meta-analysis showed that in a sample of 175.000 people, an average salt intake greater than 5 g per day increased the risk of stroke by 1.2 in exposed versus unexposed patients. In other words, a reduction, even a small one, of the risk on the whole population could lead to a considerable number of cardiovascular events avoided [34].

The family history or genetic factors of hypertension are associated with UBP in our study. This result is similar to those reported by the sub-Saharian countries [18, 21, 35–37]; this could be explained by a demotivation of the patients to fight the disease.

Stress, a mental health problem, can lead to certain unhealthy mechanisms such as excessive consumption of alcohol, smoking or binge eating or even forgetting to take medication, which is a risk of increasing blood pressure and allows compromising efforts to control blood pressure [38]. Thus, the literature reports stress as a risk factor associated with UBP [39], however this did not emerge in our survey.

Numerous studies have reported overweight and obesity as risk factors associated with UBP [23, 39]. Each ten kilograms excess in comparison to the ideal weight results in a rise of 3 mmHg of the SBP and 2 mmHg for the DBP [40]. This association is more marked in case of so-called visceral android obesity. We found the same results in our study.

Non-adherence to the drug, marked by the lack of the drug prescription monitoring would be associated with UBP. This same finding has been reported in Sudan, Ethiopia, Cameroon and Tanzania [41–44]. This could be due to 1. insufficient stocks of anti-hypertensive drugs at PHCF that can last several months, 2. expensive anti-hypertensive drugs in pharmacies, 3. low income per household, 4. non-prioritization of RAMED beneficiaries during the distribution of drugs at the PHCF, 5. lack of family support, 6. unsatisfactory relationship between the patient and the care team, and 7. irregular medical follow-up of hypertensive patients.

Therapeutic inertia could be explained by: 1. an overestimation of the effect of the prescribed treatments, 2. the physician’s doubts on the adherence to the treatment, 3. the assumption by the physician that the patient does not lead a healthy lifestyle prevents the decision to intensify the treatment, 4. a lack of physician’s training to achieve therapeutic goals [45], 5. the influence of the socio-economic background of the hypertensive patient on the medical decision 6. the lack of the appropriate antihypertensive treatment at PHCF; and 7. non-adherence to the reference frames for the management of hypertensive patients.

## Limitations of the study

Our study had a few limitations, including its cross-sectional design. Investigators use this design for its speed, easiness, and cost-efficiency. Cross-sectional designs are suited to investigate chronic diseases such as hypertension; however, they have a limited ability to document the causal relationship between exposure and uncontrolled blood pressure measured at the same time. In this study, the information collected about the sodium diet was qualitative, based on the declaration of the patient, which did not quantify the salt consumed. We also acknowledged this prevarication bias when collecting data in relation to alcohol consumption, tobacco or salt, physical activity and income per household.

## Conclusion

Our study shows a high prevalence of UBP amongst subjects followed at PHCF in Meknes. Therapeutic-inertia, non-adherence to drug, non-employment, low monthly income per household, overweight/obesity, family history of hypertension and male sex were associated with UBP. Based on our findings we recommend: i) improving adherence of health professionals to the guidelines and standards of management of hypertensive patients; ii) improving adherence to the drug; and iii) promoting a healthy lifestyle.

## Supporting information

S1 Questionnaire Used for face-to-face patients’ interview and self-administered by the physician. (PDF)
