# Supplementary material for: Prevalence of uncontrolled blood pressure in Meknes, Morocco, and its associated risk factors in 2017
Source: PLoS One. 2019 Aug 9;14(8):e0220710. doi: 10.1371/journal.pone.0220710 (PMC6688818; doi:10.1371/journal.pone.0220710)
Supplement: S1 Questionnaire — (PDF) [file pone.0220710.s001.pdf]

Questionnaire of hypertensive patients

Survey Information

Survey location: -------------------------- Date of survey:/__//__/ /__/__/ /__//__/

dd mm yy

Interviewer ID /____//____/ (Initial of interviewer + initials of primary care health center)

Time of the interview: /__//__/ /__//__/ Hour Minute

Participant ID Number /__//__/ /____//____/ (patient order number + interviewer ID)

Consent has been read: /__/ yes /__/ no

If NO, END

Consent has been obtained, orally or written form: /__/ yes /__/ non

If NO, END

Additional Information that may be helpful

Contact phone number where possible

The information collected through this document will be treated with the utmost confidentiality and will be subject to the rules of ethics relating to the respect of patient privacy and medical confidentiality.

Demographic Information

Gender: Male /__/ Female /__/ **Age:** /___/ years **or** date of birth /__//__//__/__/

Residence Area: Urban /__/ Rural /__/

Marital status: single /__/ married /__/ divorced /__/ widowed /__/

Education: Can read and write /__/ cannot read nor write /__/

Level of education: illiterate /__/ primary /__/ secondary /__/ university /__/

Occupation: without /__/ with /__/ if with, specify:--

Monthly income per household in Dhs: □≤1500 □ >1500-≤2000 □ >2000- 3000 □>3000-4999 □ ≥5.000

Health insurance coverage: Private insurance /__/ RAMED /__/ without /__/

Risk factors

Blood pressure duration: ________ years

Circumstance of discovery of high blood pressure

Accidental □ Routine exam □ Systematic exam □ Complication □Associated disease □ Other, to specify:…………………………………………………………………………………………….

General knowledge about blood pressure			To be completed at the end
**Symptoms**	yes	no	
Headache			
Auditory whistling			
Blurred vision (feeling of flies in front of the eyes)			
Dizziness			
Palpitation (fast heart rate)			
Difficulty breathing			
Epistaxis (bleeding nose)			
Hematuria (presence of blood in the urine)			
Œdema			
**Complications**	yes	no	
Stroke			
Heart attack			
Kidney damage			
Eye damage			
**Compliance with preventive guidelines**	yes	no	
Dietary and hygiene rules			
- loss of weight			
- ↓consumption of alcohol			
- ↓Tobacco			
Physical activity			
Stress avoidance			
Dietary compliance			
Compliance with treatment			
Taking medication that can lead to an increase in hypertension			
Self-measurement of blood pressure and regular medical (monitoring) follow-up			
**Variables associated with lifestyle / behaviors**	yes	no	
-Tobacco			If yes, specify : current smoker former smoker<br>How many years have you stopped smoking?:<br><br>Number of cigarettes per day:

-Alcohol	◯	◯	If yes, specify : ◯ Current drinker ◯ Former Drinker<br>For how many years :<br><br>Quantity of glasses per day:
-Sedentarity	◯	◯	To be completed at the end
			How many hours the patient remains seated or lying down:<br>…………………………………………………………
-Physical activity of more than 10 minutes duration (affiliate) (?) that has resulted in increased heart rate (moderate-vigorous)<br><br>* Physical activity of more than 10 minutes duration in a row to move from one place to another, resulting in increased heart rate<br><br>* Physical activity of more than 10 minutes leisurely that has led to an increase in heart rate (walking, running, fitness.)	◯<br><br>◯<br><br>◯	◯<br><br>◯<br><br>◯	If yes how many times per week and how much time<br>---<br><br>If yes : ◯ Low ◯ Moderate ◯ Vigorous<br>---<br><br>If yes : ◯ Low ◯ Moderate ◯ Vigorous<br>---<br><br>If yes : ◯ Low ◯ Moderate ◯ Vigorous

NB: low refers to intensity if the activity is carried out only one day or during less than 2 hours a week, while intense if it will be carried out at least for 4 days / week or more than 7 hours / week. it will be called moderate for the rest

**Stress**			◯ no ◯ Moderately ◯ a lot
**Dietary compliance**	**yes** ◯	**no** ◯	To be completed at the end
-Consumption of salt	◯	◯	If yes : ◯ semi salty ◯ salty
-Difficulty to follow the diet	◯	◯	Why? If yes, please explain :
**Family history of blood pressure**	◯	◯	If yes : ◯ mother ◯ father ◯ sister ◯ brother ◯ grandparent
**Comorbidity**	**yes**	**no**	
-Heart disease	◯	◯	If yes, type:--
-Diabetes	◯	◯	If yes type : ◯ type 1 ◯ type 2
-Dyslipidemia	◯	◯	
-Chronic renal failure	◯	◯	
**Treatment**	yes	no	Name of the drug

(Pharmaceutical Trade Mark) drug<br>- Monotherapy	○	○	○ Angiotensin II converting enzyme inhibitor ○ thiazide diuretic ○ beta-blocker ○ calcium channel blocker ○ Angiotensin II receptor antagonist
-Dual therapy	○	○	○ Angiotensin II converting enzyme inhibitor ○ thiazide diuretic ○ beta-blocker ○ calcium channel blocker ○ Angiotensin II receptor antagonist
-triple therapy	○	○	○ Angiotensin II converting enzyme inhibitor ○ thiazide diuretic ○ beta-blocker ○ calcium channel blocker ○ Angiotensin II receptor antagonist
Generic drug (?)	○	○	○ Angiotensin II converting enzyme inhibitor ○ thiazide diuretic ○ beta-blocker ○ calcium channel blocker ○ Angiotensin II receptor antagonist

Treatment time in months : --

**Compliance with treatment**	○	○	To be completed at the end
Did you forget to take your medication this morning?			○ yes ○ no
Have you been out of medication since the last consultation?			○ yes ○ no. If yes : Did you: ○ buy it from the pharmacy ○ take another drug ○ remained without ○ other
Have you had to take your treatment late compared to the usual time?			○ yes ○ no
Have you ever missed your treatment because some days because of your memory lacking			○ yes ○ no
Have you ever missed treatment because some days you feel that your treatment is doing more harm than good			○ yes ○ no
Do you think you have too many (tablets) pills to take?			○ yes ○ no

**Frequency of blood pressure self-monitoring**	○ yes ○ no	To be completed at the end
Do you measure your home blood pressure?	○ yes ○ no	If no, why :<br>○ No instrument of measure<br>○ other to specify

Relationship with the care system

Do you go to(get an appointment with) a doctor for your tension	○ yes ○ no	If yes, does it belong to the sector ○ public ○ private
Frequency of medical appointments	○ once a month ○ every 2 months ○ every three months ○ every six months ○ never ○ just if I fell myself bad	
What is the distance between your house and	○ <6 km ○ between 6 and 10 km ○ >10	

health center	km	
Time taken to reach the health center	○ less than 30 min ○ between 30 mn and 1 h ○ more than one hour	
transportation mode	○ walk ○ Chariot ○ Taxi ○ Bus ○ car ○ bicycle	
Availability of the drug for blood pressure	○ yes ○ no	
**Patient doctor relationship**		To be completed at the end
How long does the doctor allow you during the medical appointment (consultation) in term of time (in minutes)		
Do you discuss with your doctor your personal concerns/issues about hypertension?	○ yes ○ no	
Do you (feel the desire of) expect your doctor to better understand you?	○ yes ○ no	
Are you setting blood pressure goals?	○ yes ○ no	
Do someone (we) call you when you miss your appointment	○ yes ○ no	
**Do you have an association for hypertensive**		○ yes ○ no

Anthropological measurement (?)

	Systolic, in mmHg	Diastolic, in mmHg
1th measure, right arm		
1st measure, left arm		
2nd measurement at the high pressure arm		
3rd measurement at the high pressure arm		
**Mean**		

Weight (kg): _____________ Height (cm): _____________

Hip circumference (cm): ____________ Waist (cm):_____________

Blood pressure during last three visits (mmHg):_________, __________, __________

Follow-up review

ECG: ○ yes ○ no Result:

Electrolytes or ions (sodium, potassium): ○ yes ○ no Result:

Creatinine blood test: ○ yes ○ no Result:

Fasting blood glucose or glycated Hemoglobin (HbA1c): ○ yes ○ no Result:

Lipid profil: ○ yes ○ no

Total Cholesterol: ○ yes ○ no Result:

C-HDL: ○ yes ○ no Result:

C-LDL: ○ yes ○ no Result:

Triglycerides: ○ yes ○ no Result:

Doctor's questionnaire on blood pressure

Survey information
Survey location: --------------------------------- Date of survey:/__//__/ /__/__/ /__//__/
dd mm yy

Patient identify
/__//__/ /____//____/ (patient order number + interviewer ID)

Weight (kg) : ____________ (Size) Height (cm) : ____________
Hip circumference (cm) : ___________ Waist (cm):____________
Blood pressure during last visit (mmHg):____________

The information collected by means of this document will be treated with the utmost confidentiality and are subject to the rules of ethics relating to the respect of the medical confidentiality.
Treatment modification

Management of associated cardiovascular risk factors: (Nicotine) Replacement ☐ therapy for weaning tobacco☐, Weight loss☐, control of diabetes☐ , taking a statin☐ , taking a cardioaspirin☐

Please check (tick) all the corresponding box that apply

**Modified** treatment	Check box
Blood pressure in poorly controlled consultation	◯
Self-measurement made by poorly controlled patient	◯
Poorly tolerated treatment	◯
Prescription of generic drugs	◯
Trial of a new drug	◯
Prescription of a fixed combination	◯
Simplification of the medical prescription	◯
Decrease in the cost of prescription	◯
Follow-up of Ministry of Health program guidelines	◯
Other(s) to be specified	◯

**Unmodified treatment**	Check box
Poor compliance with treatment by the patient	◯
Controlled blood pressure at the previous visit	◯
Reluctance of the patient to take a new medicine	◯
Cost of important drugs	◯
White coat hypertension syndrome	◯
Etiological blood test in progress	◯
Difficulty in controlling blood pressure despite several therapeutic modifications	◯
Recent change of treatment (less to 4 weeks)	◯
Multiple drug intolerance syndrome	◯
Current intake of drugs that can affect the BP (anti inflammatory, corticoids, estrogen-progestative association ...)	◯
Management of associated cardiovascular risk factors (smoking cessation, weight loss, diabetes balance, statin)	◯
Chronic alcoholic poisoning	◯
Lack of time in consultation to explain the new treatment	◯
Lack of motivation for the patient	◯
Recent emotional event	◯
Others	◯
